# Usability of technological tools to overcome language barriers in healthcare– a scoping review

**DOI:** 10.1186/s13690-025-01543-1

**Published:** 2025-02-25

**Authors:** Annika Kreienbrinck, Saskia Hanft-Robert, Alina Ioana Forray, Asithandile Nozewu, Mike Mösko

**Affiliations:** 1 Department of Medical Psychology, University Medical Center Hamburg-Eppendorf, Martinistraße 52, Building West 26, 20246 Hamburg, Germany; 2 Department of Applied Human Sciences, Magdeburg-Stendal University of Applied Sciences, Stendal, Germany; 3 Faculty of Political, Administrative and Communication Sciences, Center for Health Innovation, Babeș-Bolyai University, Cluj-Napoca, Romania; 4 Department of Community Medicine, Discipline of Public Health and Management, Iuliu Hațieganu University of Medicine and Pharmacy, Cluj-Napoca, Romania; 5 Department of Psychology, Stellenbosch University, Stellenbosch, South Africa

**Keywords:** Communication, Language barrier, Language proficiency, Translation technology, Language discordance, Medical settings, Healthcare communication, Multilingual consultations, Medical translation, Digital communication tool

## Abstract

**Introduction:**

In many healthcare contexts globally, where the languages of care providers and service users do not match, miscommunication can lead to inaccurate diagnoses and subpar treatment outcomes. The development and use of technological tools to overcome language barriers are increasing, but usability and evaluation of these tools vary widely.

**Objectives:**

This scoping review’s objectives are (i) to identify and describe the technological tools used in direct service user–provider communication to overcome language barriers in a healthcare setting, (ii) to identify how the usability of these tools was evaluated, and (iii) to identify the challenges and benefits of using such technological tools.

**Methods and analysis:**

The scoping review followed the JBI methodology. Studies published between January 2019 and July 2024 were identified using a search strategy with variations of the keywords “technological tools,” “language barrier,” and “health care” in the following six databases and research platforms: PubMed, PsycArticle, Scopus, EBSCOhost, ProQuest, and Web of Science. All literature on individuals using a technological tool to overcome language barriers in a healthcare context was included and exported into the screening assistant software Rayyan. The search was limited to articles written in German or English. The literature was screened twice by three independent reviewers in a blinded fashion, and all relevant data were presented in a descriptive summary.

**Results:**

Based on 16 publications, this scoping review identified 16 technological tools, categorized as fixed-phrase or machine translation apps, to overcome language barriers in a healthcare setting. Usability was assessed in 13 publications applying diverse methods, i.e., surveys, observations, and application data analysis. Technological tools hold potential as a means to address language barriers in healthcare by facilitating communication and supporting diagnostic processes. However, their usability is often constrained by challenges related to translation accuracy, accessibility, and learnability.

**Conclusion:**

Future research and policy efforts should focus on standardizing evaluation methods and diversifying development regionally, linguistically, and interdisciplinary. Rather than broadly promoting these tools, emphasis should be placed on ensuring they are reliable and efficient for their intended use to maximize their effectiveness and relevance in specific healthcare contexts.

**Supplementary Information:**

The online version contains supplementary material available at 10.1186/s13690-025-01543-1.

**Table:** 

Text box 1. Contributions to the literature
• This scoping review identifies and categorizes 16 technological tools used to address language barriers in healthcare, emphasizing their varied usability across different healthcare settings and populations.
• It highlights gaps in current research, particularly the lack of standardized usability evaluation methods, and calls for regionally and culturally inclusive tool development.
• Rather than broadly promoting these tools, the review emphasizes the importance of ensuring their reliability and efficiency to maximize effectiveness and relevance in specific healthcare contexts.
• By pinpointing challenges such as translation accuracy, accessibility, and adoption barriers, the review provides a foundation for targeted improvements in technological tool design and implementation.

## Introduction

Language barriers are common in healthcare settings around the world and can affect access to and utilization of care, as well as the course and quality of treatment [1–3]. Confusion among service users and healthcare providers about diagnosis and treatment can lead to reduced treatment adherence, worse outcomes, and premature treatment discontinuation [3–6]. Although the problem of language barriers in healthcare is well known, addressing and improving the situation still needs to be addressed in research, education, and practice. Many countries, either implicitly or explicitly, expect migrants to learn the dominant language, placing the responsibility for overcoming a language barrier on individuals rather than on the healthcare system or adopting a shared approach [7, 8].

Informal practices, such as relying on family members, multilingual staff, gestures, or “receptive multilingualism”, are common in healthcare settings lacking alternatives [9–12]. While understandable, these methods can lead to translation errors or omissions of critical information [5, 11–13]. Professional interpreters are a preferred solution when providers and users lack a shared language, but their availability is often limited by legal, funding, or qualification gaps, as well as provider attitudes or shortages of interpreters for less common languages [14–17].

Technological tools can help overcome language barriers in healthcare contexts when professional interpreters are unavailable [11, 18]. These tools and applications (apps) include online dictionaries, pre-translated fixed-phrase apps, and machine translation (MT), such as Google Translate (GT) [18–22]. A 2021 study found that GT correctly conveyed the basic meaning of discharge instructions in 82% of cases, though accuracy varied by language, with errors leading to nonsensical translations in some instances [20]. In 2023, GT’s accuracy in translating mental health content was assessed, revealing challenges with medical terms and high error rates in Arabic, Persian, and Romanian, while Turkish showed fewer errors despite being a low-resource language [21]. A 2024 study compared GT and ChatGPT for translating pediatric discharge instructions, finding they performed well for Spanish and Portuguese but had significant issues with Haitian Creole [22]. Specialized medical translation tools also exist. In Germany, a fixed-phrase translation app was tested in the infirmary of a refugee arrival center and enabled users to select symptoms via pre-translated phrases in 13 languages, with 76% finding it easy to use [18]. Another fixed-phrase translation app for emergency services achieved a usability score indicating good effectiveness [23].

Previous reviews have examined the development, acceptability, and effectiveness of translation apps in healthcare settings, especially for overcoming language barriers with migrants and linguistically diverse patients. A systematic review covering data up to 2019 found that effective apps often use speech rather than text, offer features like interpreter-call options and conversation-saving capabilities, and can reduce anxiety, consultation time, and reliance on interpreters. However, limitations in fostering therapeutic relationships were noted [19].

A scoping review (2013–2022) found that interpretation tools improved communication and reduced provider frustration, with high acceptance among service users and providers. Fixed-phrase tools were particularly well received and valuable for symptom communication, though some apps were initially time-consuming, and interactions were occasionally prolonged. Overall, 93% of participating providers reported improved communication [24].

A systematic review (2012–2023) on mobile translation apps in Australian medical imaging showed benefits for routine and out-of-hours care, reducing frustration and enhancing care quality. Challenges included reliability, accuracy, particularly with free-text input, and concerns about privacy and practicality. These apps were deemed, by study participants, suitable for low-risk conversations, easing demand on interpreters [25].

An overarching observation is that there are currently no binding standards for assessing quality criteria and indicators such as usability, user behavior, and translation quality when testing or using technological tools to overcome language barriers in healthcare [19, 25, 26]. This review uses the umbrella term usability to map the existing body of evidence to address this lack of binding standards.

Usability refers to how effectively and efficiently users, in this context, both service providers and users, can interact with and use technological tools to facilitate communication despite language barriers [27]. Therefore, usability is the extent to which an application is easy to learn, intuitive to use, and enables users to perform their tasks accurately and efficiently. One of the dimensions of usability is learnability, which refers to the ease with which users, including providers and service users, can quickly understand and engage with the application’s interface and features. In this specific context, efficiency pertains to the app users’ speed when performing tasks and interacting with the application. Effectiveness focuses on the app’s fundamental purpose of enabling coherent communication despite language barriers in healthcare, which includes assessing the accuracy of translation and interpretation to ensure clear and precise communication. Satisfaction is determined by the users’ perceptions of the application’s aesthetics, ease of use, and overall experience. Accessibility refers to the app’s capacity to accommodate users with varying technical skills and potential impairments in an inclusive manner [27]. Surveys, qualitative interviews, user feedback, observational studies, and objective performance metrics are common tools used to gather evidence [13, 19, 23–26].

A scoping review methodology is particularly fitting in this case due to the rapid technological advancements and the continuous growth of research in this area. This approach allows for a broad and systematic mapping of existing literature since 2019, capturing a wide range of relevant studies and providing a comprehensive overview of translation apps and tool developments. Additionally, the scoping review enables us to address specific research questions by synthesizing newly published reviews and identifying gaps, trends, or emerging themes not fully covered in previous studies, ensuring a more up-to-date and holistic understanding of the field [19, 24, 25].

The aims of this review are (i) to identify and describe the technological tools used in direct service user–provider communication to overcome language barriers in a healthcare setting, (ii) to identify how the usability of these tools is being evaluated, and (iii) to identify the challenges and benefits of using technological tools.

## Methods

### Protocol and registration

The scoping review was registered on the Open Science Framework (OSF, https://osf.io/xepsq/) during the protocol development stage. An a priori protocol was developed using the Johanna Briggs Institute (JBI) guidelines for scoping reviews [28]. The final version of the protocol is available from the corresponding author upon request or can be found as an open-access publication [29]. This scoping review followed the five steps recommended by the JBI: (1) protocol development, (2) search for relevant studies, (3) study selection, (4) charting evidence, and (5) data synthesis [28, 30]. The findings are reported according to the Preferred Reporting Items for Systematic Reviews and Meta-Analyses extension for Scoping Reviews (PRISMA-ScR) checklist provided in the additional files (see additional file 1) [31].

### Eligibility criteria

#### Participants

The scoping review includes literature in which individuals communicate directly with each other while experiencing language barriers in a healthcare context. Publications were excluded if the communication barrier was due to other reasons, such as deafness or other physical and mental impairments. Similarly, studies were excluded if a person (interpreter) was used to overcome the language barrier, as the focus was on using technological tools that allow direct and immediate communication. Otherwise, no studies were excluded based on participants’ sex, age, or other sociodemographic factors.

#### Concept

Studies that identify and describe the technological tools used in direct and immediate service user–provider communication to overcome language barriers by optimally assessing indicators, such as usability, were included. Studies were excluded if they presented tools or aids based only on print material or if the focus of the application was not on communication between individuals (e.g., multilingual eMental Health apps).

#### Context

The context of this scoping review was defined as medical consultations and healthcare settings where people communicate directly and immediately with each other to receive healthcare services.

### Types of sources and search strategy

The search strategy aimed to identify published, unpublished, and gray literature in online databases and research platforms. Based on the results of a preliminary search (databases: PubMed, PsycArticle), a search strategy was developed using variations of the keywords “technological tools,” “language barrier,” and “healthcare” in the following databases: PubMed, PsycArticle, Scopus. These databases were chosen because they allowed us to search many publications in the biomedical, life sciences, and psychosocial fields, as well as in the social sciences and humanities. The research platform EBSCOhost was used, where specific linguistic, healthcare, and psychology databases could be searched. ProQuest Dissertations & Theses and Web of Science were also used. The search strategy, including all identified keywords and index terms, was adapted for each database and information source included. A comprehensive search strategy is provided (see additional file 2). In addition, the references of the included studies were manually searched for suitable publications that may not have been identified in the databases by the search strategy applied.

This scoping review includes most sources and, therefore, study designs, apart from reviews, if they fit the inclusion criteria. Study protocols were included only if they provided insights into the usability of a tool, mainly through pilot test results or plans for evaluating the tool. We will indicate when data extractions from a protocol are discussed in the results and discussion sections. Broadly thematically overlapping reviews were presented in the introduction to provide an overview of the existing research landscape. Studies had to be accessible through the databases and research platforms searched in this review to be eligible for inclusion. However, any relevant studies discovered during the screening of reference lists from the included sources of evidence were examined regardless of this criterion. Studies inaccessible in full text were excluded as abstracts typically provide limited information.

The search was limited to articles written in German or English, aligning with the researchers’ language proficiency. Studies published since 2019 were included, as the review by Thonon et al. (2021) has a broad thematic overlap and already presents findings from the literature up to 2019 [19]. The first search of databases and research platforms was performed in September 2023. Given the time interval since the initial search, an updated search was conducted in July 2024.

### Study/source of evidence selection

Following the search, all identified citations were collated and uploaded into Rayyan screening assistant software, and duplicates were removed [32]. The Rayyan software facilitated the screening of titles and abstracts, which were at least assessed twice against the inclusion criteria by the independent reviewers AK, AF, and AN in a blinded fashion. Reviewers AK, AF, and AN retrieved the remaining sources in full text, assessed them independently, and were blinded in detail. The reasons for the exclusion of sources of evidence were recorded and reported. Disagreements between the reviewers at any stage of the selection process were resolved by discussion within the three-person screening team.

### Data extraction

The data were manually extracted from the included literature via a self-developed extraction form: authors, year, country, type of publication/reference, study design, objective, participants, sample size, concept, context, method of data collection, method of data analysis, limitations, and key findings relevant to the research questions. The finalized data extraction form is provided (see additional file 3). The tool was tested, modified, and revised as necessary while extracting data from each source of evidence included. The data extraction form, method, and results were presented and discussed with the review team. Any disagreements were resolved by consensus.

### Methodological quality appraisal

Consistent with JBI guidance for scoping reviews, the methodological quality or risk of bias of the included studies was not assessed because the focus is on mapping and summarizing the literature. The aim is to provide an overview, identify gaps, and highlight emerging themes rather than critically appraise individual studies and their study rigor [28].

### Data summary and synthesis

A PRISMA-ScR flowchart and description offer an overview of the evidence search and selection process. The studies’ characteristics are summarized in narrative and descriptive form, as are findings on participant characteristics, technological tools to overcome language barriers, and key factors related to usability and other indicators. This synthesis and presentation of findings examines the technological tools available to overcome language barriers in a healthcare context, how indicators such as the tool’s usability were assessed, and what benefits and challenges were noted. The findings of this review provide an overview of the existing body of studies, identify research gaps, and thus provide guidance for future research.

## Results

### Selection of sources of evidence

**Fig. 1:**
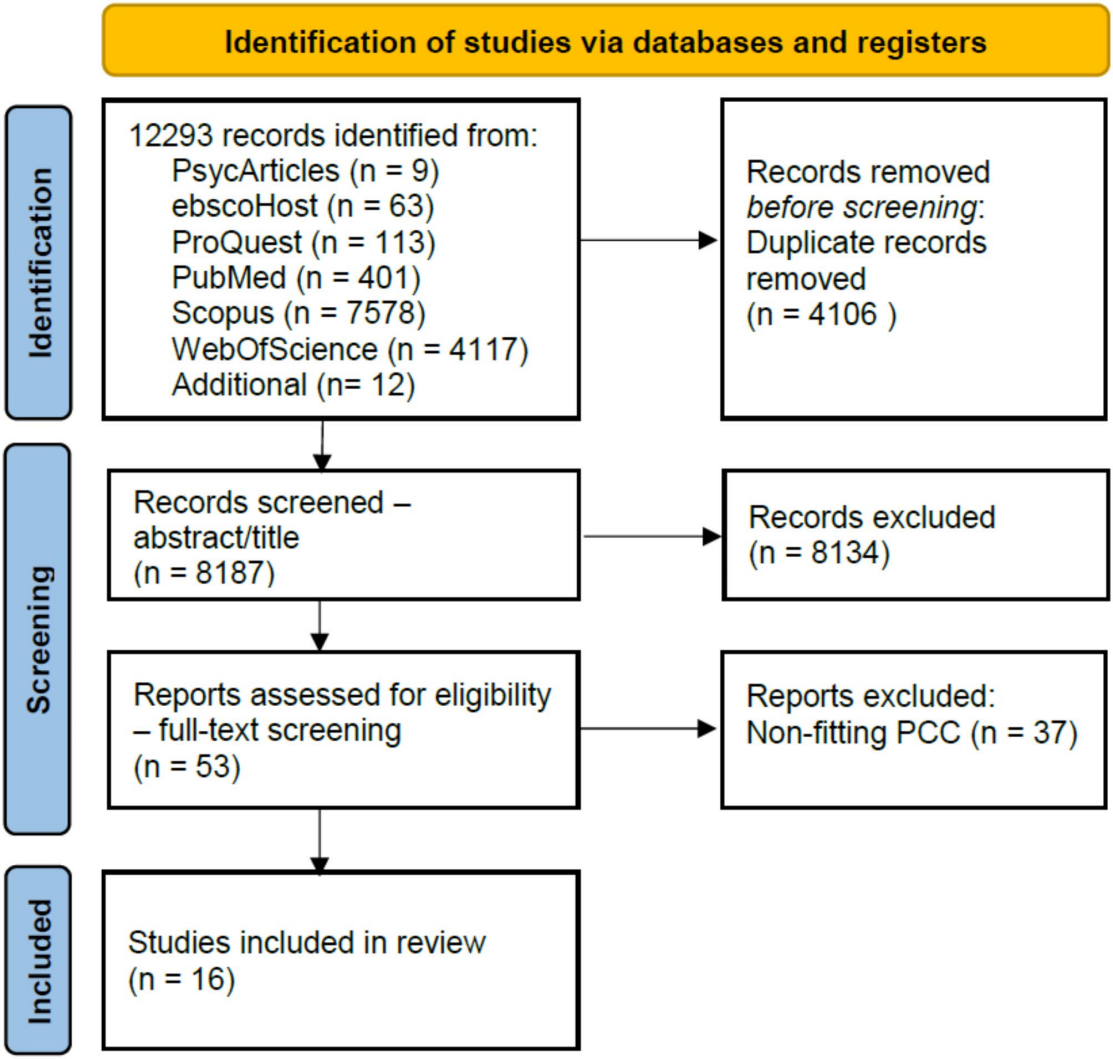
Prisma-ScR flowchart of the literature selection in the review (2019–2024)

A total of 8,187 records were retrieved from six databases and research platforms and manually searched after duplicates were removed. Following the title and abstract screening, 36 records were assessed for eligibility via a full-text screening, of which 16 studies fit the set inclusion criteria of this scoping review (see Fig. 1).

### Study characteristics

Among the 16 articles, seven originated from Germany [18, 23, 35, 37, 40–42], three from Australia [13, 26, 39], two from Belgium [33, 34], three from Switzerland [36, 38, 43], and one from France [44] (see Table 1). Five of the articles were published in 2019 [13, 26, 34–36], two in 2020 [13, 40], five in 2021 [18, 33, 37, 42, 44], two in 2022 [23, 39], one in 2023 [41], and one in 2024 [38]. The articles were published mainly in English [13, 26, 33–44]; two were published in German [18, 23]. An Australian research group is represented in three publications assessing translation apps in aged care settings [13, 26, 39]. A German research group contributed to six publications, focusing on developing and evaluating two fixed-phrase translation tools for prehospital medical care [23, 41, 42] and general practice [18, 35, 40].

The healthcare settings for the conducted or planned studies were as follows: one study worked with the term healthcare setting and no further specification [26]; four studies focused on emergency medical care [33, 36, 37, 43]; three focused on prehospital emergency medical care [23, 41, 42]; five focused on general practice [18, 34, 35, 40, 44]; one focused on primary care [38]; and two focused on nursing homes and aged-care hospital wards [13, 39] (see Table 1).

A variety of study designs were included, which can be categorized as follows: Six studies used or planned to use a quantitative approach [23, 36, 40, 41, 43, 44], while mixed-methods approaches were employed in five studies [13, 26, 34, 35, 39]. Additionally, three studies adopted qualitative designs [33, 38, 42], and two focused on usability [18, 37].

With the overarching theme of “experiencing a language barrier,” the study’s target population was people with low language proficiency [34, 36, 38, 39, 41]. Some studies have defined populations as migrants or cultural minorities [13, 26, 33, 44] and refugees and asylum seekers [18, 35, 40]. The second prominent target population was healthcare providers [13, 23, 34, 36–38, 42, 43]. Medical doctors, medical students, nurses, and paramedics were among the professional groups included in the studies.

The primary language in this context refers to the official language or the common language spoken by service providers in the studies, usually the language translated to or from. In eight studies, German emerged as the most frequently represented primary language [18, 23, 33, 35, 37, 40–42]. English was the primary language in four studies [13, 26, 39, 44]. French was included in four studies [33, 36, 38, 43], and Dutch in two studies [33, 34]. The characteristics of the included studies and protocols are presented in a table and additional file (see Table 1 and additional file 4).

**Table 1:** Characteristics of the 16 studies included in the scoping review focusing on technological tools to overcome language barriers in a healthcare setting (2019–2024)

Study Characteristics	Publications (*n* = 16)
**Year of publication**	
2019	5
2020	2
2021	5
2022	2
2023	1
2024	1
**Language of publication**	
English	14
German	2
**Study country**	
Germany	7
France	1
Belgium	2
Australia	3
Switzerland	3
**Study design**	
Mixed-methods	5
Quantitative	6
Usability	2
Qualitative	3
**Target population**	
People with low language proficiency	5
Migrants and/or cultural minorities	5
Refugees/Asylum seekers	3
Healthcare professionals	8
**Healthcare settings**	
Healthcare/not further defined	1
Prehospital emergency medical care	3
General practitioner/Medical practice	5
Nursing homes/aged-care hospital wards	2
Emergency medical care	4
Primary care	1

### Characteristics of technological tools

The included literature identified 16 technological tools to overcome language barriers, all mobile applications. Nine (56.3%) apps were developed for use in healthcare settings, and seven (43.8%) apps were developed for general language translation purposes. Three apps (18.8%) were assessed during or as part of the development process by the researchers undertaking the respective studies [18, 23, 34, 35, 40–42].

Six mobile applications (37.5%) used machine translation and were developed for general settings beyond the healthcare context. Nine tools (56.3%) used fixed phrases designed for healthcare-specific settings. One tool (6.25%) also used fixed phrases and was developed for general conversational settings (see Table 2).

Most technological tools support between one and 20 languages (*n* = 10; 62.5%). An equal 6.3% (*n* = 1) each support 21–40, 41–60, or 81–100 languages. Two applications (12.5%) support more than 100 languages.

Three apps (18.8%) require a monetary fee for use. Eleven tools (68.8%) are available and can be used free of monetary charge. For two apps (12.5%), no information concerning the monetary costs of the application and usage could be found in the articles.

**Table 2:** Characteristics of the 16 technological tools analyzed in the scoping review (2019–2024)

Application Characteristics	Apps (*n* = 16), n(%)
**Type of translation technology**	
Machine translation/AI (general)	6 (37.5)
Fixed-phrase (healthcare specific)	9 (56.3)
Fixed-phrase (general)	1 (6.3)
**Supported languages**	
1–20	10 (62.5)
21–40	1 (6.3)
41–60	1 (6.3)
61–80	1 (6.3)
81–100	1 (6.3)
> 100	2 (12.5)
**Costs/Fee required**	
Yes	3 (18.8)
No	11 (68.8)
No information in the publication	2 (12.5)
**Specific development for healthcare settings**	
Yes	9 (56.3)
No	7 (43.8)

### Evaluation of usability

Among the 16 publications included, usability evaluations were conducted in 13 studies, employing diverse methods to assess various dimensions of usability, such as satisfaction, efficiency, learnability, effectiveness, and accessibility. Two studies outlined plans for future usability assessments as part of their protocols [35, 44], while one study did not assess usability as it focused on other aspects of multilingual healthcare interactions [33]. The protocols are included in this section as they provide insights into planned evaluation methodologies and rationales. However, they are excluded from subsequent analysis of benefits and challenges, as the studies have not yet been conducted.

A variety of methods were employed to assess usability aspects. Table 3 provides an overview of the evaluation methods used, their frequency, the usability dimensions they assessed, and examples. Surveys, including standardized tools like the system usability scale (SUS) [45] and AttrakDiff [46], were the most frequently used method applied in 13 studies [13, 18, 23, 26, 34, 36–44]. These surveys primarily focused on dimensions like satisfaction, efficiency, and learnability, capturing users’ overall experience, ease of use, and perceived utility of the tools. Four studies used or planned the SUS [13, 23, 43, 44], while AttrakDiff was applied in one study to explore hedonic and pragmatic qualities [23].

Observations and field notes were employed in eight studies, allowing researchers to evaluate effectiveness, efficiency, and accessibility through real or simulated interactions [13, 23, 26, 34, 37–39, 41]. These methods provided insights into how tools performed in practice, including barriers and enablers to their implementation.

App data analysis was conducted in six studies, focusing on efficiency and effectiveness through metrics such as length of session times, task completion rates, and error frequencies [18, 23, 37, 40–42].

Focus groups, used in two studies, offered qualitative insights into satisfaction and accessibility [13, 44]. These discussions revealed user perceptions, challenges, and benefits, particularly among linguistically diverse service users and providers.

Feature analysis was conducted in one study, focusing on interface usability, language support, and offline functionality [26]. The study critically examined tools’ compatibility with healthcare tasks and adaptability to diverse clinical contexts.

The results are visually presented in Table 3.

**Table 3:** Overview of the methods used to assess usability in the studies included in this scoping review (2019–2024)

Evaluation method	Frequency (*n*)	Examples of usability outcomes assessed	Usability aspects assessed
Surveys (e.g., SUS, AttrakDiff)	13	Ease of use, satisfaction, hedonic and pragmatic qualities	Satisfaction, Efficiency, Learnability
Observations & field notes	8	Barriers and enablers, real-world implementation	Effectiveness, Efficiency, Accessibility
App data analysis	6	Task completion rates, session times, error frequencies	Efficiency, Effectiveness
Focus groups	2	User perceptions, challenges, and preferences	Satisfaction, Accessibility
Feature analysis	1	Interface usability, offline functionality	Learnability, Accessibility

A notable finding from the scoping review is that many studies employed multi-method approaches to assess usability, integrating qualitative and quantitative methods to provide a comprehensive evaluation. For instance, Panayiotou et al. (2020) combined focus groups and surveys to explore user satisfaction and accessibility, capturing subjective perceptions and structured appraisals of translation tools [13]. Similarly, Hwang et al. (2022) utilized observations during real-world interactions and staff surveys to assess the acceptability and effectiveness of translation apps, complementing user-reported feedback with contextual insights [39]. Müller et al. (2022) employed the System Usability Scale (SUS) alongside app usage data analysis to evaluate efficiency, satisfaction, and learnability in prehospital settings, effectively merging standardized scales with objective performance metrics [23]. These multi-method approaches illustrate the variety of techniques used to capture the diverse aspects of usability.

### Benefits and challenges of usability

The reviewed studies provided numerous insights into the benefits and challenges of using technological tools to overcome language barriers in healthcare. According to six studies, fixed-phrase tools improved communication efficiency and diagnostic accuracy, particularly in emergency and prehospital settings where structured interactions were common [23, 36, 37, 41–43]. Additionally, five studies noted that fixed-phrase tools enabled basic communication in multiple languages, making them a cost-effective alternative to professional interpreters, particularly for uncommon languages and dialects [13, 23, 26, 42, 43]. Additionally, three studies indicated that fixed-phrase tools could fill critical gaps when professional interpreters were unavailable, assisting with assessment, treatment, discharge planning, and other essential care activities [26, 38, 43]. Some apps featured visual and interactive elements, as shown in two studies, to help explain abstract concepts, build emotional connections, and reduce the need for repeated explanations from service providers [34, 38]. Documenting features, such as recording conversation history, were identified as useful in two studies, as they enhanced information retention and supported follow-up care [41, 42].

Six studies mentioned that machine translation tools offered flexibility in covering a broader range of languages [26, 36–39, 43]. One study reported that service providers achieved their consultation goals in 82.7% of encounters using machine translation tools. However, only 53.8% of users were satisfied with these interactions, reflecting ongoing challenges with usability and translation accuracy [38]. Across four studies, both fixed-phrase and machine translation tools received high usability ratings, with participants noting their ease of use and effectiveness in capturing essential information [13, 18, 23, 38].

Despite these benefits, the reviewed studies also identified several challenges. In eight studies, translation accuracy and reliability were reported as major issues, particularly with machine translation tools. Frequent errors compromised diagnostic decisions and confused service users and providers [13, 23, 26, 38, 39, 42, 43]. Usability challenges, such as difficulties with real-time speech recognition in noisy environments and issues with dialectal expressions, were noted in five studies, highlighting technical limitations [13, 26, 36, 38, 39]. According to three studies, the lack of flexibility and specific phrases for specialized medical contexts reduced fixed-phrase apps’ effectiveness, especially in healthcare disciplines requiring highly technical terminology [38, 42, 43].

Concerns about empathy and cultural adaptability were raised in two studies. These studies noted that translation apps may reduce empathy in service user-provider interactions by failing to convey emotional nuances and relational subtleties. Furthermore, the inability of these tools to adapt culturally, as human interpreters may be able to, was seen as a limitation in ensuring meaningful and sensitive communication [13, 37]. Concerns about data security and accessibility were noted in four studies, with barriers including internet dependency, perceived risks to personal health data, and subscription costs [23, 26, 42, 43]. Additionally, four studies found that using translation apps sometimes extended interaction times, as users needed to familiarize themselves with the tools and explain their operation during consultations. This challenge was particularly pronounced in high-pressure settings such as prehospital emergency care, detracting from the efficiency gains otherwise associated with these tools [34, 38, 39, 41].

## Discussion

### Summary of evidence

This scoping review sought to identify and describe the technological tools used in direct service user–provider communication to overcome language barriers in a healthcare setting, identify how the usability of these tools is being evaluated, and identify the challenges and benefits of using technological tools. It included 16 studies published between 2019 and 2024, predominantly conducted in high-income countries, mainly Europe and Australia. The diversity of healthcare settings examined, ranging from emergency care to general practice and aged-care wards, demonstrates the broad applicability of these tools. However, the specific demands of different settings may impact tool usability. Tools that performed well in structured and time-sensitive environments, such as emergency care, may require adaptation for more nuanced interactions, such as those in general practice or mental health care.

The studies employed various designs, including mixed methods, quantitative, qualitative, and usability studies, reflecting the complexity of evaluating these tools across diverse contexts. While this multifaceted approach provides valuable insights, the methodologies’ variability limits the findings’ comparability. Target populations included service users with limited language proficiency, e.g., migrants, refugees, asylum seekers, and service providers such as medical students, paramedics, and physicians. Including multiple perspectives is vital for evaluating usability. However, several studies firmly focused on the perspectives of healthcare providers, potentially underrepresenting the unique challenges that service users may face, such as understanding healthcare-specific terminology.

### Implications for practice

The findings reveal that technological tools can enhance communication between service users and providers, particularly in emergency and prehospital settings where time-sensitive communication is critical. Fixed-phrase apps provide pre-translated and structured content, improving diagnostic accuracy and communication efficiency and addressing immediate language barriers in resource-constrained scenarios [42, 43]. However, these tools may inadvertently prolong treatment time due to the need for user familiarization, while their limited phrase libraries reduce flexibility and effectiveness in more specialized medical contexts [26, 39, 42]. This trade-off between immediacy and flexibility highlights further need to evaluate these tools’ applicability across diverse contexts. As Kiblinger et al. (2024) noted, while fixed-phrase tools are effective for low-complexity interactions, they fail to capture nuances required in more complex consultations [24].

Machine translation tools offer broader linguistic applicability but are prone to errors that could jeopardize service user care, particularly in translating medical terminology and preserving contextual nuance [26, 36, 39, 43]. Delfani et al. (2023) emphasized significant challenges in mental health settings, where such errors could compromise care [21]. Similarly, Brewster et al. (2024) highlighted inaccuracies in MT-generated discharge instructions, reinforcing the idea that these tools should supplement professional interpreters rather than replace them, particularly in critical or culturally sensitive interactions [22]. Emergency and prehospital care settings often face unexpected language barriers under time and resource constraints, where these tools can create a base level of communication [34, 38, 41]. Therefore, integrating MT tools into healthcare must be approached cautiously, ensuring they complement existing communication systems rather than detract from the structured use of professional interpreters.

Barriers to adopting these tools, including concerns around data security, internet dependency, and cost, present additional challenges, particularly in resource-limited settings [13, 23, 26, 42, 43]. Rigorous data protection policies and developing offline-compatible tools are essential to foster trust and accessibility. For instance, Spechbach et al. (2019) stressed the importance of secure systems tailored to healthcare contexts [43]. Addressing these barriers requires a balance between accessibility and safeguarding service users’ privacy, alongside ethical considerations regarding consent and usage.

Training healthcare providers on these tools’ appropriate use and limitations is critical to minimizing risks. Providers must be equipped with the skills to assess the quality and appropriateness of technological solutions in real time and use them responsibly in clinical practice [13, 19, 22]. Brewster et al. (2024) stressed that healthcare providers risk misinterpreting the tools’ outputs without proper training, potentially leading to errors or miscommunication [22]. Training programs should focus on equipping providers with skills to assess tool accuracy and appropriateness in real-time. Additionally, Taylor et al. (2024) recommended that embedding clear organizational guidelines into clinical workflows is crucial for ensuring alignment with broader patient safety and communication goals [25]. Policymakers and healthcare stakeholders should prioritize supporting the development and evaluation of these tools based on robust evidence. This includes ensuring efficiency and tailoring tools to specific clinical scenarios where they are most effective [13, 22, 25]. Furthermore, organizational guidelines should clearly define when these tools are appropriate, ensuring their integration into clinical workflows aligns with broader patient safety and communication goals [22]. Tailoring tools to specific scenarios and adopting evidence-based evaluation frameworks will enhance their utility and integration.

### Implications for research

Expanding Research into Specialized Fields.

While much of the existing research on translation tools has focused on emergency and general practice, evaluating their applicability in other specialized fields, such as mental health, is a critical area for future exploration [13, 21]. Mental health communication often involves complex and emotionally sensitive exchanges that require greater cultural and contextual adaptability. Research by Delfani et al. (2023) on GT’s performance in mental healthcare highlighted significant challenges in conveying medical terminology and emotional nuance, particularly in Arabic and Persian translations [21]. These findings emphasize the need for customized translation engines for specialized fields supported by human oversight to address quality issues and ensure that these tools meet the unique demands of diverse healthcare contexts [13, 21]. Additionally, Panayiotou et al. (2020) found that fixed-phrase translation tools also struggled with high-stakes interactions due to their inability to account for emotional subtleties and nuanced phrasing [13].

Standardizing Usability Evaluation Criteria.

The development and implementation of standardized usability evaluation criteria are vital for advancing research and practice in this domain. Current studies, while valuable, often adopt diverse methodologies for evaluating usability, limiting the comparability of findings and generalizability of recommendations [42] For example, Kiblinger et al. (2024) noted that usability evaluation methods for point-of-care interpretation tools varied significantly, making it difficult to assess their effectiveness across different settings, while Brewster et al. (2024) highlighted the importance of frameworks that assess both usability dimensions and broader healthcare outcomes, such as reducing medical errors and improving service user-provider communication [22, 24]. Consistent evaluation frameworks would support robust comparisons and evidence-based recommendations for tool integration into healthcare settings [13, 22, 42].

### Integrating cultural sensitivity and empathy

A critical limitation of current technological tools is their inability to adapt to cultural nuances or convey empathy, which are essential components of effective communication, particularly in sensitive fields like mental health [13, 21]. Taylor et al. (2024) and Spechbach et al. (2019) emphasized the need for culturally adaptive designs to enhance user trust and acceptance [25, 43]. Panayiotou et al. (2020) found that participatory design processes involving diverse stakeholders can yield tools that better address users’ emotional and relational needs [13].

### Diversify languages and regions

Most existing studies are concentrated in high-income countries, limiting their applicability to resource-constrained regions with unique healthcare challenges. Expanding research to underrepresented regions is critical to addressing global healthcare inequities and ensuring tools are adaptable for diverse linguistic and cultural contexts [24, 25]. They could also explore the role of offline tools and low-cost solutions to address barriers such as internet dependency and hardware availability [25, 26, 39].

Incorporating diverse languages and regions in research would ensure that tools are inclusive and effective across various linguistic and cultural settings. Prior studies have emphasized the need for multilingual training data to improve the accuracy and usability of machine translation tools. For example, Brewster et al. (2024) highlighted how machine translation platforms, including ChatGPT and Google Translate, performed inconsistently across languages, with significant quality disparities observed in less-resourced languages like Haitian Creole compared to Spanish or Portuguese [22]. Similarly, Delfani et al. (2023) emphasized challenges in translating mental healthcare information into languages such as Arabic and Romanian due to limitations in domain-specific terminology and syntactic structure [21]. These findings reinforce the necessity of diverse linguistic representation in training data.

### Multidisciplinary collaboration

The involvement of multidisciplinary teams in designing and evaluating these tools is essential for achieving comprehensive and user-centered innovation. Panayiotou et al. (2020) and Kiblinger et al. (2024) demonstrated the value of participatory methods that involve healthcare workers, linguists, and developers [13, 24]. Moreover, feedback loops for continuous improvement were mentioned as essential to maintaining adaptability in rapidly evolving healthcare environments. Iterative feedback loops, as noted by Taylor et al. (2024), can ensure continuous improvement and adaptability to evolving healthcare needs [25]. This iterative process could address critical gaps such as integrating dynamic updates to translation databases based on user feedback and evolving medical terminologies. Spechbach et al. (2019) recommend that collaboration must also address ethical concerns, including data privacy and translation biases, to ensure these tools align with broader healthcare equity goals [43].

### Strengths and limitations

The review ensures methodological rigor and transparency by adhering to the JBI framework and PRISMA-ScR guidelines. We adhered to blinded and independent screening and data extraction processes. We included diverse methodological designs, including mixed methods and quantitative studies, to get a comprehensive overview. This approach enabled a multifaceted understanding of the tools’ usability across healthcare contexts. Moreover, including target populations representing service users (e.g., migrants, refugees, and asylum seekers) and service providers (e.g., physicians, paramedics, and medical students) let us capture multiple perspectives.

However, several limitations must be acknowledged. First, choosing the scoping review methodology inherently limits the critical appraisal of the quality or risk of bias in the included studies. As such, while the breadth of evidence is mapped effectively, the depth of evaluation regarding methodological rigor and study validity is limited. Additionally, the broad inclusion criteria and diverse study contexts might contribute to heterogeneity, making it challenging to draw definitive conclusions about the effectiveness or best practices of the tools identified. These limitations are inherent to the scoping review approach, which aims to provide an overview rather than a detailed critical synthesis. Future systematic reviews or meta-analyses could complement these findings by evaluating the effectiveness and quality of the tools using more stringent criteria. Second, the review’s restriction to publications in English and German likely introduces publication bias, as it may exclude relevant studies published in other languages, mainly from low- and middle-income countries where language barriers are also prevalent. This bias could result in overrepresenting findings from high-income regions and underestimating challenges and solutions tailored to other world regions. Third, the geographic concentration of studies in high-income countries, mainly Europe and Australia, creates a geographic bias, potentially overlooking the unique healthcare challenges in other world regions. The review also faces limitations related to the rapid pace of technological advancement. Some tools in the review may already be outdated, while newer technologies might not yet be represented in the literature.

Additionally, the focus on tools for direct service user-provider communication may have excluded hybrid tools with broader functionalities, such as service user education and documentation, which could offer complementary insights. The variability in usability evaluation methods across studies further complicates comparisons, underscoring the need for standardized evaluation frameworks to ensure consistency and generalizability. Another limitation is the lack of long-term impact assessments. Most included studies focus on short-term usability and immediate outcomes, leaving gaps in understanding the sustainability and scalability of these tools. Furthermore, while the review notes barriers such as internet dependency, hardware availability, and digital literacy, these factors could have been analyzed in greater depth to explore their impact on tool adoption in resource-limited settings.

Finally, while emergency care and general practice are represented, the lack of available literature on specialized fields like mental health highlights a gap in existing research. Communication in such fields often requires greater cultural and contextual adaptability, emphasizing the need for future studies to address these complexities and inform the design of tools that are both effective and inclusive for use in specialized healthcare interactions.

## Conclusion

This scoping review aimed to identify and describe the technological tools used to overcome language barriers in a healthcare setting, how the usability of these tools was evaluated, and the challenges and benefits of using such technological tools. Fixed-phrase tools presented better efficiency in structured scenarios but faced challenges such as limited phrase libraries and reduced flexibility in specialized contexts. Machine translation tools offered broader linguistic coverage and flexibility but encountered issues such as accuracy and contextual nuance.

Usability assessments across the included studies varied widely; the findings underscore the need for context-specific adaptations, comprehensive training for healthcare providers, and, if integrating the tools, using them to supplement rather than replacements for professional interpreters, particularly in complex or culturally sensitive interactions.

Rather than simply promoting the broader use of these applications, the emphasis should be on ensuring that they are reliable and efficient for the specific situations in which they are intended to be used. Future research should prioritize the development of standardized usability evaluation frameworks, expand studies to include underrepresented geographic regions and languages, and explore the long-term impacts of these tools on healthcare outcomes and system efficiency to achieve this. Additionally, interdisciplinary collaboration among developers, linguists, healthcare professionals, and other stakeholders is essential to ensure these tools are culturally sensitive, user-centered, and effective in practice.

Policymakers and stakeholders should focus on creating equitable access to these tools, supporting the development of offline-compatible and cost-effective solutions, and establishing clear guidelines for their integration into healthcare systems.

## Electronic supplementary material

Below is the link to the electronic supplementary material.

Supplementary Material 1

Supplementary Material 2

Supplementary Material 3

Supplementary Material 4

## Data Availability

No datasets were generated or analysed during the current study.

## Abbreviations

App: Application
GT: Google Translate
JBI: Joanna Briggs Institute
MT: Machine Translation
OSF: Open Science Framework
PCC: Population, Concept and Context
PRISMA-ScR: Preferred Reporting Items for Systematic Reviews and Meta-Analyses
SUS: System Usability Scale
