# Supplementary material for: Usability of technological tools to overcome language barriers in healthcare– a scoping review
Source: Arch Public Health. 2025 Feb 25;83:52. doi: 10.1186/s13690-025-01543-1 (PMC11852517; doi:10.1186/s13690-025-01543-1)
Supplement: Supplementary file 2 — Supplementary Material 2 [file 13690_2025_1543_MOESM2_ESM.docx]

## Additional file 4: Selected characteristics of the included studies focusing on technological tools for overcoming language barriers in healthcare settings (2019-2024)

| **Author (Year of publication)** | **Country of origin** | **Study design/method** | **Objective** | **Population** | **Concept: App translator type/app name** | **Usability assessed (y/n)** | **Key results regarding usability aspect evaluation** |
| --- | --- | --- | --- | --- | --- | --- | --- |
| Cox & Maryns (2021) | Belgium | Ethnographic | Analyze multilingual strategies in emergency department consultations - Examine the impact of ad hoc multilingual solutions on interaction dynamics | Displaced population, Immigrant patients | Fixed-phrase tool; Universal Doctor Speaker | no |  |
| deWilde et al. (2019) | Belgium | Mixed-methods | To design and implement a multilingual, mobile app to facilitate communication in language discordant face-to-face service encounters. To investigate the impact of technological mediation on information transfer in service encounters. To explore the use of technology in improving service quality and satisfaction in language discordant encounters. To assess the effectiveness of the app in enhancing interactions and satisfaction levels in simulated service encounters. To identify the need for additional training to improve service providers' trust and confidence in using technology in service encounters. | Service providers and service users experiencing language barriers | Fixed-phrase tool, dictionary; K&G app | yes | Benefits:   - Visuals in the app helped build emotional connections, clarify words, and explain abstract ideas. - The app effectively facilitated visualization of concrete objects and communication of abstract concepts. - The app’s dictionary tool provided translation, visual representation, and audio, aiding in explaining difficult concepts.  Challenges:  - Technology-facilitated service encounters lengthened visit times, reduced eye contact, and highlighted the need for additional training. - Service providers were reluctant to use the app in real settings due to insecurity and lack of tech-savviness. - Technology use interrupted eye contact, affecting natural communication flow. - Technology-facilitated encounters lasted longer with more frequent and longer pauses compared to technology-free encounters. - Reduced client-centered eye gaze was noted, though averted gaze could enhance multimodal communication depending on the provider’s skills and training. |
| Furaijat et al. (2019) | Germany | Mixed-methods study protocol | Assess usability and accuracy of digital translation tool - Investigate impact on mutual understanding between patients and GPs | Refugee patients | Fixed-phrase tool; DCAT (digital communication assistance tool) | no |  |
| Halimi & Bouillon (2019) | Switzerland | Crossover study | The study aimed to measure the impact of translation quality on communication and assess the dangerousness of incorrect translations. | Medical professionals and non-french-speaking patients | fixed-phrase: BabelDr (health care specific) // MT:Google translate | yes | Benefits   - BabelDr significantly aided doctor-patient communication in emergency settings, providing reliable output. - BabelDr's domain-trained system improved accessibility and communication with Arabic-speaking patients in medical settings. - The system’s speech recognition and translation capabilities ensured accurate communication between French-speaking medical staff and Arabic-speaking patients.  Challenges:  - Meaningless translations by technology can lead to repetitive questioning in emergency medical dialogues, hindering rapid patient management. - Google Translate may produce dissonant meanings, impacting effective communication between medical staff and patients. - Mistranslations by Google Translate were considered dangerous, conveying contrary meanings and posing risks in medical communication. - Discrepancies and meaningless translations can result in doctors asking multiple questions to ensure patient understanding, potentially slowing down communication. |
| Herrmann-Werner et al. (2021) | Germany | Usability study | Evaluate the use of a speech-to-speech language translation app in a simulated physician-patient situation. - Assess the perceived usability, helpfulness, and meaningfulness of the language translation app. | Medical students | iTranslate Converse | yes | Benefits:  - Students found the language translation app very useful for emergency situations and brief conversations. - The app was considered more beneficial in hospital contexts than in ambulatory settings. - A highlighted advantage was the app's ability to create dialogue transcripts, especially in the upgraded paid version. - The app was seen as cost-effective compared to human translators, particularly useful for translating uncommon languages and dialects.  Challenges:  - Students were concerned about translation errors potentially jeopardizing diagnostic decisions. - They feared the app could decrease empathy in patient-physician communication and raised concerns about data protection and technical reliability. - The inflexibility of LTAs compared to human interaction was noted as a disadvantage, particularly in adapting to cultural differences and communication nuances. |
| Hudelson et al. (2024) | Switzerland | Exploratory study | Explore factors that may hinder or facilitate communication when using voice to voice machine translation | Healthcare service providers, service users | Machine Translation with voice-to-voice function: Microsoft Translate, Pockettalk |  | Benefits:  - Patients appreciated the ease of reading translations, enhancing their understanding without needing repeated explanations from healthcare professionals or interpreters. - The app was seen as a tool to promote patient autonomy, reduce dependency on others for translation, and improve communication with healthcare providers. - Health professionals found the app useful for consultations involving factual information, simple exams, and partial language barriers, making it a valuable alternative to telephone interpreters in noisy environments. - Both patients and healthcare professionals recognized the app's potential to enhance communication autonomy and ensure confidentiality.  Challenges:  - Lack of familiarity and practice with the app made communication more difficult for both patients and healthcare professionals. - Communication could take longer than with an interpreter if time was taken during the consultation to explain the app. - Speech recognition problems and translation errors occurred in various scenarios, such as disfluent speech, non-standard dialects, and technical issues like internet connection problems. |
| Hwang et al. (2022) | Australia | Mixed-methods | Explore the use of mobile translation apps in aged-care hospital wards. - Assess the effectiveness and acceptability of translation apps in overcoming language barriers in healthcare settings. | Older Australians with limited English proficiency | CALDassist & TalkToMe fixed phrase (health care specific) // google translate (MT) | yes | Benefits:  - Applications improved communication between healthcare workers and patients, aiding in retrieving information during emergencies. - They provided reassurance to patients and created excitement or novelty when using the translation app. - The apps enhanced patient-staff interactions and helped overcome language barriers in healthcare settings.  Challenges:  - The translation app did not always improve rapport, sometimes causing frustration for both staff and patients. - Patients were sometimes confused by the app, especially if they didn’t understand, which could be a drawback. - Inaccuracy of translation, particularly with Google Translate, was a noted issue. - The apps lacked specific phrases for allied health disciplines in their phrase libraries. - Difficulty in understanding older patients' responses was a challenge when using phrase-based translation apps. |
| Kleinert et al. (2021) | Germany | Usability study | Evaluate usability and efficiency of an anamnesis app - Test and optimize the app for broader medical use | Refugees & asylum seekers | Fixed-phrase: DCAT (digital communication assistance tool) | yes | - 76% of users rated the application as "easy to use," indicating a positive user experience. - 65% of users successfully entered their main complaints using the app, demonstrating its effectiveness in capturing essential information. - The app was not prone to errors from online translations, ensuring accurate communication and data entry. |
| Müller et al. (2020) | Germany | Nonrandomized controlled pilot study | Develop a digital communication assistance tool (DCAT) - Obtain medical history from foreign-language patients | Refugee patients | Fixed-phrase: DCAT (digital communication assistance tool) | yes | - The DCAT app was widely accepted by refugee patients during pilot testing, showing high acceptance and usability. - The DCAT app was piloted with 36 refugee patients, recording between 5 and 12 symptoms per patient according to the ICPC-2, with an average time of 1.7 minutes per symptom.  Challenges:  - Need for extensive understanding of written language and the ability to use a touchscreen keyboard. - Difficulties in instant translation, especially with dialectal expressions, limited the use of free text entry in the app. - Pictograms were often misunderstood during testing, leading to their reduced use in the intervention. |
| Müller et al. (2022) | Germany | Quantitative pilot study | Evaluate the usability of the smartphone app in the pilot study - Assess the perception of the app by rescue workers who have actively used it with patients | Paramedics | Fixed-phrase: Aidminutes | yes | - The application was rated overall good in usability and attractiveness, especially considering its role as a complex work tool in the EMS context. - Experienced paramedics who actively used the application rated it significantly better, indicating a positive impact on usability. - The app facilitated basic communication in 18 languages through 600 pre-recorded phrases in 20 languages, demonstrating its practical and valuable functionality.  Next Development Goal:  - Improving usability by identifying content that was difficult to find or frequently used phrases, and making adjustments in the phrase groupings. |
| Müller et al. (2023) | Germany | Nonrandomized controlled pilot study | Evaluate the efficacy and feasibility of a multilingual app for paramedics. - Gain insights into the app's impact on paramedic-patient communication. | Patients with limited German language proficiency, paramedics | Fixed-phrase tool: Aidminutes | yes | Benefits:  - The application supported 18 languages, facilitating communication with foreign-language patients. - Paramedics perceived a higher overall quality of communication when using the app with patients with limited German proficiency. - Sensitivity analyses confirmed the app's positive impact on communication, showing consistent results across different models.  Negative:  - App usage led to longer emergency scene times, extending them by 6-7 minutes. |
| Noack et al. (2021) | Germany | Participatory design | Develop a digital communication tool for paramedics - Overcome language barriers in prehospital emergency care | Paramedics | Fixed-phrase tool: Aidminutes | yes | Benefits:  - The application included 600 fixed phrases in 18 languages, effectively overcoming language barriers in prehospital emergency care. - It featured phrases for medical history-taking, consent requests, and additional information, making it comprehensive and versatile. - The app was designed for rapid use, with phrases available audibly or as text, categorized for easy access and adapted to various situations. - It included a function to document patient responses and conversation history, enhancing communication and information retention during emergency care.  Challenges:  - Potential ambiguities in translations could be unintentionally misleading. - Grouping comprehensive content so that all phrases could be easily found was a challenge. - The need for phrase review by proficient individuals in the respective languages and medical professionals before and after translation was highlighted. - Anticipating future uses of the technology was recommended to address potential challenges and opportunities after deployment. - The app's content and communication approach needed adaptation for the complex, volatile, and rushed nature of prehospital emergency care, indicating a need for specific adjustments to suit rescue operations. |
| Panayioutou et al. (2019) | Australia | Mixed-methods | Evaluate language translation apps in health care settings - Determine suitability for everyday conversations in health care settings | People without proficient English from non-English speaking countries - Culturally and linguistically diverse individuals in Australia | Fixed-phrase (Medical): CALDassist; TalkToMe; Canopy speak, medibabble translator, UniversalDoctor // fixed-phrase (general): TripLingo // MT: google translate; microsoft translator; naver papago, SayHi Translate | yes | Benefits:  - Apps enable everyday conversations in healthcare settings. - They provide a solution when professional interpreters are unavailable, supporting key aspects of care like assessment, treatment, discharge planning, and obtaining consent. - Apps help with orientation cues and conveying essential care needs, such as identifying pain or toileting needs. - They are suitable for situations where an interpreter isn't necessary, offering support for essential communication in healthcare settings.  Challenges:  - Some apps, like iTranslate, iTranslate Voice, and Speak and Translate, require monthly subscriptions, which led to their exclusion from the study. - Challenges include the need for caution regarding translation accuracy and cultural suitability, which were beyond the study's scope but crucial for effective use in healthcare. - The study emphasized the importance of evaluating language translation apps in healthcare, especially in everyday clinical conversations and with specific cohorts like older non-English speaking patients. |
| Panayioutou et al. (2020) | Australia | Multi-method | To understand attitudes and perceptions of older people and healthcare workers towards translation technology in healthcare settings. - To explore the potential use of mobile translation apps for everyday healthcare communications | Older people from culturally and linguistically diverse backgrounds - Healthcare workers in Australia | CALDassist & TalkToMe fixed phrase (health care specific) // google translate (MT) | yes | Benefits:  - Participants rated TalkToMe and Google Translate (GT) highly for ease of use and overall satisfaction. - Healthcare professionals (HCPs) also reported high perceived ease of use and satisfaction with TalkToMe and GT.  Challenges:  - Issues identified include accuracy of translations and phrases, potential technological learning curves, risk of mistranslation in high-risk conversations, and the inability to verify translation accuracy. - Real-time voice-to-voice translation apps like Google Translate struggled with word recognition due to background noise or different dialects. - Participants from the Chinese community consultation expressed confusion and dissatisfaction with the app translations. - Instances of inaccurate and confusing translations negatively impacted the effectiveness of the applications. |
| Spechbach et al. (2019) | Switzerland | Crossover study | Assess if BabelDr can be used for diagnostic interviews in emergency settings. - Investigate if speech is a useful modality in the tool. | French-speaking doctors | Fixed-phrase: BabelDr | yes | - Doctors successfully reached correct diagnoses using BabelDr, a speech-enabled fixed-phrase translator, in emergency conditions. - The study confirmed that speech functionality is crucial for such tools, highlighting its usefulness in emergency settings. - BabelDr, like other fixed-phrase translators, allowed doctors to freely ask questions and match recognition results to predefined sentences, enhancing usability. - The system was designed to be easily adaptable to new languages and situations, meeting hospital needs and ensuring integration across different services. - The translation process follows standard procedures, performed online with translation memory, focusing on languages important to Geneva University Hospitals. - Apps like BabelDr reliably collect information from patients when no interpreter is available, enabling doctors to confidently reach a diagnosis, even though some may feel constrained by the system. - BabelDr served as an alternative to machine translation, offering reliable information collection. |
| Thonon et al. (2021) | France | Stepped wedge randomized controlled trial – study protocol | Develop a multilingual electronic tool (app) for health providers. - Evaluate the acceptability and impact of the app. | Migrants with a language barrier | Not developed yet | No |  |
