# Supplementary material for: Usability of technological tools to overcome language barriers in healthcare– a scoping review
Source: Arch Public Health. 2025 Feb 25;83:52. doi: 10.1186/s13690-025-01543-1 (PMC11852517; doi:10.1186/s13690-025-01543-1)
Supplement: Supplementary file 3 — Supplementary Material 3 [file 13690_2025_1543_MOESM3_ESM.docx]

### Additional file 2: Search strategy used to identify studies on technological tools for overcoming language barriers in a healthcare setting (2019-2024)

PubMed: Search conducted on September 22, 2023

| Search | Query | Results |
| --- | --- | --- |
| #1 | ("mobile applications"[MeSH Terms] OR "Translations"[MeSH Terms] OR "Smartphone"[MeSH Terms] OR "translation tool*"[Title/Abstract] OR "translation device*"[Title/Abstract] OR "translation app*"[Title/Abstract] OR "translation application*"[Title/Abstract] OR "google translate"[Title/Abstract] OR "computer*"[Title/Abstract] OR "smartphone*"[Title/Abstract] OR "cell phone*"[Title/Abstract] OR "mobile phone*"[Title/Abstract] OR "mobile technolog*"[Title/Abstract] OR "mHealth"[Title/Abstract] OR ("telemedicine"[MeSH Terms] OR "telemedicine"[All Fields] OR "ehealth"[All Fields]) OR "technology-enabled"[Title/Abstract] OR "machine translation"[All Fields] OR "machine learning"[All Fields]) AND ("communication barrier*"[MeSH Terms] OR "Translating"[MeSH Terms] OR "Transients and Migrants"[MeSH Terms] OR "Refugees"[MeSH Terms] OR "limited language proficiency"[Title/Abstract] OR "language barrier*"[Title/Abstract] OR "multilingual*"[Title/Abstract]) AND ("mental disorders"[MeSH Terms] OR "Mental Health"[MeSH Terms] OR "mental disorders"[MeSH Terms] OR "psychiatric care"[Title/Abstract] OR "psychother*"[Title/Abstract] OR "psych*"[Title/Abstract] OR "mental"[Title/Abstract] OR "psychology"[Title/Abstract] OR "psychiatric unit"[Title/Abstract] OR "health care"[Title/Abstract] OR "hospital"[Title/Abstract] OR "primary care"[Title/Abstract] OR "hospital care"[Title/Abstract] OR "emergency room"[Title/Abstract] OR "emergency service*"[Title/Abstract] OR "health service"[Title/Abstract]) | 569 |
| #2  Filters for  Years: 2019-2023  Language: German, English | (("mobile applications"[MeSH Terms] OR "Translations"[MeSH Terms] OR "Smartphone"[MeSH Terms] OR "translation tool*"[Title/Abstract] OR "translation device*"[Title/Abstract] OR "translation app*"[Title/Abstract] OR "translation application*"[Title/Abstract] OR "google translate"[Title/Abstract] OR "computer*"[Title/Abstract] OR "smartphone*"[Title/Abstract] OR "cell phone*"[Title/Abstract] OR "mobile phone*"[Title/Abstract] OR "mobile technolog*"[Title/Abstract] OR "mHealth"[Title/Abstract] OR ("telemedicine"[MeSH Terms] OR "telemedicine"[All Fields] OR "ehealth"[All Fields]) OR "technology-enabled"[Title/Abstract] OR "machine translation"[All Fields] OR "machine learning"[All Fields]) AND ("communication barrier*"[MeSH Terms] OR "Translating"[MeSH Terms] OR "Transients and Migrants"[MeSH Terms] OR "Refugees"[MeSH Terms] OR "limited language proficiency"[Title/Abstract] OR "language barrier*"[Title/Abstract] OR "multilingual*"[Title/Abstract]) AND ("mental disorders"[MeSH Terms] OR "Mental Health"[MeSH Terms] OR "mental disorders"[MeSH Terms] OR "psychiatric care"[Title/Abstract] OR "psychother*"[Title/Abstract] OR "psych*"[Title/Abstract] OR "mental"[Title/Abstract] OR "psychology"[Title/Abstract] OR "psychiatric unit"[Title/Abstract] OR "health care"[Title/Abstract] OR "hospital"[Title/Abstract] OR "primary care"[Title/Abstract] OR "hospital care"[Title/Abstract] OR "emergency room"[Title/Abstract] OR "emergency service*"[Title/Abstract] OR "health service"[Title/Abstract])) AND (2019:2023[pdat]) | 257 |

Most recent search: June 10, 2024 (updated filter for years: 2023-2024)
