# Supplementary material for: Usability of technological tools to overcome language barriers in healthcare– a scoping review
Source: Arch Public Health. 2025 Feb 25;83:52. doi: 10.1186/s13690-025-01543-1 (PMC11852517; doi:10.1186/s13690-025-01543-1)
Supplement: Supplementary file 4 — Supplementary Material 4 [file 13690_2025_1543_MOESM4_ESM.docx]

### Additional file 3: Data extraction form for studies evaluating technological tools to overcome language barriers in a healthcare setting (2019-2024)

|  |  |  |  |  |  |  |  | Population | | |
| --- | --- | --- | --- | --- | --- | --- | --- | --- | --- | --- |
| # | Author(s) | Year of publication | Country of origin | Title of source | Type of publication | Study design | Objecitve | Target population | Sample size | Sampling method |

| Concept | | | | | |
| --- | --- | --- | --- | --- | --- |
| Overcoming language barrier | Direct/immediate communication | Type of technological tool | App name | Supported languages | Primary language(s) translated to/from |

| Concept | | | | | | | Context | |
| --- | --- | --- | --- | --- | --- | --- | --- | --- |
| Outcomes measured | Usability measured | Learnability | Efficiency | Effectiveness | Satisfaction | Accessibility | Heathcare | Specific description |

| Methods | |  |  |  |  |
| --- | --- | --- | --- | --- | --- |
| Data collection | Data analysis | Limitations of studies | Key findings relating to RQs | DOI/Full reference | Notes |
